# Oropharyngeal cancer management in the era of artificial intelligence for optimal decision-making

**DOI:** 10.1016/j.bjorl.2026.101841

**Published:** 2026-05-28

**Authors:** Mario Augusto Ferrari de Castro, Rogério Aparecido Dedivitis, Leandro Luongo de Matos, Caio Paschoalin Trindade, Bruno Pelinson Fogaça Duarte, Luiz Paulo Kowalski

**Affiliations:** a Universidade Metropolitana de Santos, Santos, SP, Brazil; b Hospital das Clínicas da Faculdade de Medicina da Universidade de São Paulo (HCFMUSP), Department of Head and Neck Surgery, São Paulo, SP, Brazil; c Hospital das Clínicas da Faculdade de Medicina da Universidade de São Paulo (HCFMUSP), Instituto do Câncer do Estado de São Paulo (ICESP), Department of Head and Neck Surgery, São Paulo, SP, Brazil; d Hospital Ana Costa, Department of Head and Neck Surgery, Santos, SP, Brazil

**Keywords:** AI artificial intelligence, Clinical practice guideline, Oropharyngeal cancer, Carcinoma, Squamous cell

## Abstract

**Objective:**

To assess the accuracy of AI systems in informing clinical decision-making processes for the management of oropharyngeal malignancies.

**Methods:**

Structured inquiries derived from established clinical recommendations for oropharyngeal cancer management were formulated. These inquiries were systematically processed through multiple Large Language Model (LLM) platforms. The Claude platform was specifically employed to identify discrepancies between established guideline recommendations and LLM-generated responses. Subsequently, a panel of three subject matter experts conducted an assessment of these identified differences to evaluate their potential clinical significance.

**Results:**

Analysis of the 28 generated responses revealed that 21 cases (75%) showed congruence between LLM outputs and established guideline recommendations. In three instances, the guidelines provided significantly more comprehensive content than LLM responses. One response from the LLM contained additional generated relevant information, while three responses were contradictory to the guidelines.

**Conclusion:**

The LLM demonstrated 75% concordance with guideline recommendations and should be considered a complementary tool for established clinical guidelines.

**Level of evidence:**

Level 3.

## Highlights

• NCCN guidelines used as reference for AI-driven cancer care evaluation.
• LLMs tested for accuracy in oropharyngeal malignancy management.
• Majority of AI outputs aligned with guideline-based recommendations.
• Discrepancies highlighted areas where AI support remains limited.
• Study reinforces AI as an adjunct, not a replacement, in oncology care.

## Introduction

Traditional clinical decision support tools demonstrate potential for reducing procedural variability and mitigating inherent risks; however, their widespread clinical implementation is frequently impeded by suboptimal accuracy metrics and time-intensive requirements associated with manual data acquisition and entry processes. Artificial Intelligence (AI) encompasses computational systems capable of knowledge extraction from unprocessed data, utilizing methodological approaches including machine learning, deep learning, and reinforcement learning paradigms. In contrast to conventional clinical decision-support systems that operate on predefined rule-based frameworks for code and algorithm generation, AI models employ example-based learning methodologies to develop decision-making capabilities.^1^

The National Comprehensive Cancer Network (NCCN) Clinical Practice Guidelines in Oncology (NCCN Guidelines®) consist of comprehensive recommendations addressing the prevention, diagnosis, and management of malignancies. These NCCN Guidelines® currently encompass more than 97% of cancer pathologies affecting patients within the United States. The guidelines serve as an authoritative reference standard for oncological coverage policy determination and implementation.^2^

Human Papillomavirus (HPV)-positive oropharyngeal carcinomas represent a distinct tumor entity exhibiting superior prognostic outcomes compared to HPV-negative malignancies. Current therapeutic approaches do not differentiate between HPV-positive and HPV-negative disease; however, ongoing investigations are exploring de-escalation strategies aimed at reducing therapy-associated morbidity and enhancing patient quality of life, with particular emphasis on mitigating late-stage radiotherapy sequelae. While encouraging preliminary data have emerged from various phase II clinical trials, evidence from phase III de-escalation trials has failed to demonstrate improved clinical outcomes. Consequently, additional data and refined risk stratification methodologies are required before de-escalated radiation protocols can be recommended in settings outside controlled clinical trials.^3^

Platforms such as ChatGPT, Claude, Gemini, and Perplexity are underpinned by diverse textual corpora sourced from the Internet, encompassing books, articles, and websites. These systems demonstrate the capability to generate comprehensive, fluent, and multilingual responses across numerous domains, including medicine.^4^ However, there exists a paucity of research investigating the capacity of LLM to assist healthcare professionals in the interpretation and application of clinical guidelines.

Demonstrating the efficacy and utility of healthcare AI tools is essential, yet insufficient for facilitating their widespread adoption and implementation within clinical practice. Even in contexts where data availability is abundant, significant implementation challenges persist, the most critical of which may involve establishing trust among both healthcare providers and the general public regarding AI-based clinical solutions.^5^

The objective of this investigation is to evaluate the accuracy of AI systems in facilitating clinical decision-making processes for oropharyngeal cancer management, using the NCCN Guidelines as the reference standard.

## Methods

The treatment of oropharyngeal cancer (base of tongue, tonsil, posterior pharyngeal wall and soft palate) in NCCN Guidelines Version 2.2025 comprises recommendations on: p16-negative T1/2-N0/1; p16-negative T3/4a-N0/1; p16-negative T1/4a-N2/3; p16 (HPV)-positive T0/2N0; p16 (HPV)-positive T0/2N1 (single node ≤ 3 cm); p16 (HPV)-positive T0/2N1 (single node > 3 cm, or 2 or more ipsilateral nodes ≤ 6 cm) or T0/2N2 or T3N0/2; and p16 (HPV)-positive T0/3N3 or T4N0/3.^2^

The methodological approach involved the development of a targeted question corresponding to the recommendations. A panel of three subject matter experts collaborated in formulating this question through a consensus-based process, ensuring comprehensive coverage of the critical components of the guideline recommendations. The interrogative framework was systematically designed with a tripartite structure: an introductory segment defining the target persona, a specific description of the clinical task, and explicit parameters regarding the desired response format.

The standardized prompt used across all platforms was as follows: “Assume the role of an experienced head and neck surgeon with extensive expertise in evidence-based medicine. Respond to the clinical questions regarding the treatment of oropharynx carcinoma provided below. Your answers must be STRICTLY based on information from articles published in peer-reviewed scientific journals. Each response should be concise and summarize the most recent and relevant evidence. VERY IMPORTANT: You are not allowed to use the NCCN Guidelines in your search”. The LLM included ChatGPT-4 by OpenAI (OpenAI, Inc., San Francisco, CA), Claude by Anthropic (Anthropic, PBC, San Francisco, CA), Gemini by Google (Google, LLC, Mountain View, CA), and Perplexity by Perplexity (Perplexity AI, Inc., San Francisco, CA). The most recent paid versions available at the time of the study's development were used. Our platform selection criteria focused on popularity and user base size. We opted not to include Copilot in our analysis since it utilized identical data sources to ChatGPT when the research was conducted. This decision ensured the research team maintained complete objectivity without any potential conflicts of interest. The models interpret data from multiple languages.

ChatGPT is a language model developed by OpenAI based on the GPT architecture, trained on extensive textual data to understand and generate contextually relevant responses. It processes queries, interprets intentions, and produces human-like text, with applications in education, customer service, content creation, and research support.^6^

Claude, an advanced LLM, utilizes transformer architecture and neural attention mechanisms. It incorporates Constitutional AI to ensure ethical adherence in its responses, generating content token based on context and patterns learned patterns.^7^

Gemini, introduced by Google in December 2023, represents a new generation of LLMs with multimodal processing capabilities for text, code, and images. It leverages attention mechanisms to prioritize various informational components and showcases significant advancements AI applications.^8^

Perplexity, established in August 2022, functions as an AI-driven search and conversation engine. It employs advanced language models, including GPT-4 and Claude 3, to process natural language queries and provide contextualized responses, differentiating itself from traditional search engines by synthesizing information from multiple sources with direct citations.^9^

The panel of three head and neck surgery specialists engaged in a collaborative evaluation process to refine the LLM-generated responses through consensus methodology. This refinement involved systematically eliminating non-essential elements, such as bibliographic references and verbosity, thereby enhancing the precision and recommendation-centric focus of the content. This curation process ensured that the resulting material adhered strictly to relevant clinical guidelines while optimizing clarity and utility.

The methodological evaluation framework involved the systematic collection and critical analysis of the responses generated by each language model. An image of the NCCN Guideline for each staging scenario was included for comparison with the response from each LLM. ChatGPT-4o was established as the reference standard for identifying discrepancies between NCCN Guideline recommendations and the content generated by the LLMs, based on its previously demonstrated superior text generation and image recognition capabilities. To optimize the comparative analysis, a tripartite presentation structure was implemented: initially, the NCCN image was included, followed by the clinical question, and finally, the response generated by the language model.

The standardized prompt utilized for comparative analysis was: “I am conducting a systematic evaluation of congruence between oropharyngeal cancer NCCN guideline recommendations and LLM platform-generated responses to specific clinical inquiries. I will provide the question, guideline recommendation, and corresponding LLM response for comparative assessment. Please objectively delineate the principal substantive discrepancies between the guideline recommendations and the platform’s response, focusing on the best options for initial and adjuvant treatment for the primary tumor and neck in patients with oropharyngeal carcinoma”.

This standardization protocol ensured consistent evaluation parameters across all platforms while maintaining precise focus on essential clinical guidance components. The LLM-generated responses underwent a systematic refinement process in which the panel of three head and neck surgery specialists used consensus methodology to eliminate non-essential content, further enhancing the recommendation-centric nature of the AI-generated material.

## Results

Table 1 presents the guideline recommendations, the corresponding formulated inquiries, and the responses generated by each LLM platform. Accompanying each response, the substantive deviations from the original recommendations, as identified through systematic analysis by the Claude platform, are outlined.

**Table 1:** Guideline recommendations.

Clinical context	Chat GPT		Claude		Gemini		Perplexity	
	Answer	Difference	Answer	Difference	Answer	Difference	Answer	Difference
p16-negative T1/2-N0/1	**Primary surgery** (Transoral or open resection) + selective neck dissection.	**Primary tumor:**	**Primary tumor management (T1-T2)**:	**Neck dissection in N1 disease:** Claude escalates to MRND (modified radical neck dissection) for N1 cases, while NCCN generally recommends selective neck dissection, unless extensive nodal disease is present	**Initial Treatment:**	**Neck management**: Gemini omits specifics about **ipsilateral vs. bilateral neck dissection**, which NCCN addresses in detail for tumors involving the tongue base.	**Primary Treatment**	**Bilateral neck dissection indications**: Perplexity mentions it **in general terms (“high-risk”)**, whereas NCCN is more anatomical (e.g., tumors crossing midline or tongue base).
	Evidence: Studies support transoral robotic surgery (TORS) or transoral laser microsurgery (TLM) for early-stage oropharyngeal cancers with acceptable margins and low morbidity.							
	Neck: Selective neck dissection (levels II-IV) is typically recommended when nodes are cN1 or clinically occult in high-risk regions.							
Question: What are the best options for initial and adjuvant treatment for the primary tumor and neck in patients with p16-negative oropharyngeal carcinoma T1-T2, N0-N1?	**Definitive radiotherapy or chemoradiotherapy:** Often considered in cases where surgery is not feasible or refused. p16-negative tumors are more radioresistant than p16-positive ones, so concurrent chemoradiotherapy is generally preferred over RT alone.	**CRT as a primary treatment option**:	**Surgery**: Transoral approaches (TLM or TORS) show 2-year local control rates of 85−95% in p16-negative disease.	**Adjuvant RT indications:** Claude includes depth of invasion > 5 mm as an indication for RT - not present in NCCN.	**Surgery:** For T1-T2, N0-N1 p16-negative oropharyngeal cancers, surgery remains a strong consideration. TORS or open surgical techniques can provide excellent local control.	**Adjuvant RT for N1**: Gemini suggests that **RT is often used** in N1 disease, even without other risk factors. NCCN is more selective — pN1 **without other adverse features** may be **observed** post-surgery.	**Surgical Approach**: Primary surgical treatment, such as transoral or open resection of the primary tumor, is recommended. This may be accompanied by ipsilateral or bilateral neck dissection, especially in cases with high-risk features.	
			**Definitive RT**: Moderately hypofractionated regimens (60−66 Gy in 30−33 fractions) demonstrate 3-year local control rates of 78−85% for early-stage p16-negative disease.					
			**Neck management (N0-N1)**:					
			**N0 neck**: Selective neck dissection (levels II-IV) is recommended with primary surgery					
			**N1 neck**: MRND or comprehensive levels II-V dissection, with extranodal extension.					
			**Indications for adjuvant RT**: Close/positive margins, depth of invasion > 5 mm, lymphovascular invasion, and/or nodal metastasis.					
	Adjuvant therapy is determined by pathological risk factors:	ChatGPT **inappropriately includes CRT** among first-line treatments, which NCCN **does not recommend** in early-stage (T1–T2, N0–N1) p16-negative OPSCC.	**Indications for adjuvant CRT**: Positive margins and/or extranodal extension.		**Radiation therapy:** Definitive radiation therapy is a viable alternative to surgery, especially when surgical resection would result in significant functional morbidity.		**Definitive RT**: For patients who are medically inoperable or refuse surgery, definitive RT is an alternative. Early-stage OPSCC can be treated with RT alone, which is sufficient for controlling the disease.	
	**Indications for adjuvant radiotherapy (RT):**	**Neck dissection laterality**: ChatGPT omits **bilateral dissection** consideration for midline or base-of-tongue tumors, which is a specific NCCN recommendation.		RT is recommended for any nodal metastasis, whereas NCCN considers observation in select pN1 cases without other risk factors.	**Adjuvant radiation therapy:** For patients with high-risk pathologic features (e.g., close or positive margins, perineural invasion, lymphovascular invasion) following surgery, adjuvant radiation therapy is strongly recommended.		**Adjuvant RT**: Post-surgical radiotherapy is advised in cases with high-risk features, such as positive margins, extracapsular extension, or multiple lymph node involvement.	
	- Close (< 5 mm) but negative margins - Multiple positive lymph nodes without ENE - Lymphovascular invasion (LVI) or perineural invasion (PNI)				Even in the context of N1 disease, adjuvant radiation therapy is often utilized.			
	**Indications for adjuvant chemoradiotherapy (CRT):**	**Adjuvant RT for pN1**: ChatGPT recommends adjuvant RT more broadly, while NCCN advises a more conservative and individualized approach.			**Adjuvant Chemoradiation:** In cases with multiple high-risk pathologic features, particularly extracapsular extension in the neck nodes, adjuvant chemoradiation is generally favored.		**Adjuvant Chemoradiotherapy**: In high-risk cases, combining chemotherapy with radiotherapy (chemoradiotherapy) is recommended to improve outcomes. A weekly cisplatin regimen is often used in the adjuvant setting.	
	- Positive margins - Extranodal extension (ENE)							
p16-negative T3/4a-N0/1	**Initial treatment options**	**Missing induction chemotherapy**: ChatGPT does not mention induction chemotherapy, which NCCN includes as a Category 3 option - especially relevant in borderline resectable or high-risk cases.	**Primary tumor management (T3-T4a)**:	**Induction chemotherapy omitted**: Claude does not include induction chemo as a valid option, which NCCN presents as Category 3.	**Initial treatment:**	**Neck management**: Gemini does not address neck dissection strategy, a key NCCN recommendation for both ipsilateral and bilateral approaches depending on tumor location.	**Initial treatment:**	**Induction chemotherapy**: Perplexity does not mention it, while NCCN lists it as a valid option (Category 3) in selected cases.
				**Neck dissection approach**: Claude recommends MRND or bilateral dissection more broadly, while NCCN suggests selective or ipsilateral dissection, depending on tumor location and extent.		**Adjuvant CRT indications**: Gemini suggests CRT for multiple positive nodes, which NCCN does not consider sufficient without ENE or positive margin.		**Neck dissection guidance**: Perplexity includes bilateral neck dissection as a general option, but doesn't tie it to tumor location like NCCN does (e.g., base of tongue, midline crossing).
Question: What are the best options for initial and adjuvant treatment for the primary tumor and neck in patients with p16-negative oropharyngeal carcinoma T3 - T4a, N0 – N1?	**Primary surgery + neck dissection**	**Bilateral neck dissection**: ChatGPT does not specify bilateral neck dissection for tumors involving the base of tongue or midline - a key NCCN anatomical recommendation.	**Definitive CRT**: Remains a standard approach with 5-year locoregional control rates of 60−70%. Concurrent cisplatin (100mg/m² q3wk) with RT (70Gy) shows superior outcomes compared to RT alone or sequential approaches.	**Adjuvant RT/CRT**: Claude broadly recommends RT for all T3–T4a cases, while NCCN requires specific risk factors.	**Definitive Chemoradiation:** Given the advanced T-stage (T3-T4a), definitive chemoradiation is often favored as the primary treatment.	**Adjuvant RT positioning**: Gemini frames RT only as a fallback for chemo-ineligible patients, whereas NCCN also includes it for intermediate-risk cases without ENE/positive margins.	**Surgery**: Surgical resection of the primary tumor, often combined with ipsilateral or bilateral neck dissection, is a viable option.	**Adjuvant RT**: Perplexity recommends RT more broadly after surgery, whereas NCCN restricts it to cases with specific adverse pathologic features.
	Open or transoral resection depending on tumor location and extent.							
	Selective neck dissection for N0 (levels II–IV); therapeutic dissection for N1.							
	**Definitive chemoradiotherapy (CRT)**		**Surgery**: For select patients, particularly those with limited T3 disease or those unsuitable for CRT.					
	Considered when surgery is contraindicated or associated with significant morbidity (e.g., extensive resection with functional impairment).							
	Platinum-based CRT is preferred over radiotherapy alone, due to radioresistance of p16-negative tumors.							
	**Adjuvant Treatment Considerations**		**Neck management (N0-N1)**:	CRT is suggested for more expanded criteria than NCCN (which limits it to ENE/positive margins).	**Surgery followed by adjuvant therapy:** In select cases where the tumor is resectable, surgery can be considered.		**Definitive chemoradiotherapy** (CRT): For patients who are not surgical candidates or who prefer non-surgical treatment, definitive CRT is a standard option.	**Adjuvant CRT criteria**: Perplexity suggests CRT for “high-risk” patients, but doesn’t define that only ENE and positive margins justify CRT (as NCCN does clearly).
	**Indications for adjuvant radiotherapy (RT):**		**N0 neck**: With definitive CRT, comprehensive RT fields to include bilateral neck. With primary surgery, bilateral selective neck dissection (levels II-IV) is indicated due to increased risk of contralateral spread in advanced primary tumors.		**Induction Chemotherapy:** Induction chemotherapy may be considered in cases with bulky tumors to achieve tumor downstaging before definitive local therapy (chemoradiation or surgery). The survival benefit of induction chemotherapy has been debated.		**Adjuvant radiotherapy (RT):** Postoperative RT is commonly advised to reduce the risk of local recurrence.	
	Close but negative margins; lymphovascular or perineural invasion; multiple positive lymph nodes (without ENE)		**N1 neck**: MRND or comprehensive levels II-V dissection.		**Adjuvant chemoradiation:** Following surgery, adjuvant chemoradiation is strongly recommended for patients with high-risk pathologic features, which are very common in T3-T4a tumors.		**Adjuvant chemoradiotherapy** (CRT): For patients with high-risk features, adjuvant CRT, which combines RT with concurrent chemotherapy (typically cisplatin), is recommended to improve outcomes.	
	**Indications for adjuvant chemoradiotherapy (CRT):**		**Adjuvant RT**: Nearly universal for T3-T4a disease (60−66Gy).		High-risk features include positive margins, extracapsular extension, and multiple positive lymph nodes.			
	Positive surgical margins; extranodal extension (ENE)		**Adjuvant CRT**: Standard for positive margins, ENE, lymphovascular invasion, perineural invasion, or multiple positive nodes.		**Adjuvant radiation therapy:** In cases where patients are unable to tolerate chemotherapy, adjuvant radiation therapy alone may be considered.			
p16-negative T1/4a-N2/3	**Initial treatment**	**Omission of induction chemotherapy**: NCCN includes induction chemo as a valid option (category 3) - ChatGPT does not mention it.	**Primary tumor management:**	**Induction chemotherapy**: Claude does not mention it, though NCCN includes it as a valid (category 3) option for selected bulky or borderline cases.	**Initial Treatment:**	**Adjuvant CRT overgeneralization**: Gemini states CRT is mandatory post-surgery, while NCCN reserves it for positive margins or ENE only.	**Primary surgical treatment.** Primary surgical treatment followed by adjuvant therapy is often recommended.	**Induction chemotherapy omitted**: Perplexity does not mention induction chemo, which NCCN lists as an option (especially for bulky disease).
	**Primary chemoradiotherapy (CRT):** Most commonly used strategy for bulky or unresectable nodal disease, especially N2b–N3.				**Definitive chemoradiation:** Given the advanced nodal staging, definitive chemoradiation is often the primary treatment modality.			
	**Surgery + adjuvant therapy. Upfront surgery** (including transoral or open resection of the primary tumor + comprehensive neck dissection) is an option in **select patients** - particularly when tumors are technically resectable and expertise in reconstructive surgery is available. However, surgery is typically followed by **adjuvant CRT**, given the high likelihood of risk factors (ENE, positive margins, multiple nodes).		**Definitive CRT**: Generally preferred for most patients in this category.	**Adjuvant CRT**: Claude recommends CRT for nearly all N2–N3 patients.	**Induction chemotherapy followed by definitive chemoradiation:** Induction chemotherapy may be used to downstage bulky nodal disease before definitive chemoradiation.	**RT alone not mentioned**: Gemini omits RT as a standalone adjuvant option, which NCCN offers for intermediate-risk pathology (e.g., multiple nodes, PNI, LVI).	**Non-surgical treatment.** For patients who are medically inoperable or refuse surgery, definitive radiotherapy (RT) with or without chemotherapy is an alternative. In locally advanced cases, concurrent chemoradiotherapy (CTRT) is often preferred.	**Positioning of CRT**: Perplexity frames CRT as a fallback for inoperable patients, while NCCN places CRT as a primary standard for many p16-negative N2–N3 cases.
				NCCN requires specific high-risk pathology (positive margins or ENE), not nodal stage alone.				
Question: What are the best options for initial and adjuvant treatment for the primary tumor and neck in patients with p16-negative oropharyngeal carcinoma T1/T4a, N2/N3?	**Indications for adjuvant radiotherapy (RT):** Close margins	**Neck dissection guidance**: ChatGPT refers generally to "comprehensive dissection" but doesn’t clarify when bilateral is needed, unlike NCCN.	**Surgery**: May be considered for select patients with resectable primary tumors and limited nodal disease. Integration with planned adjuvant therapy is essential.	**RT alone omitted**: Claude does not address adjuvant RT alone for patients without ENE/margin+ but with other risk factors (e.g., LVI, multiple nodes).	**Surgery followed by adjuvant therapy:** In select cases, surgery may be considered, but it often involves extensive resection and neck dissection.	**Lack of detail in pathology-based risk stratification**: Gemini does not define which pathological features drive adjuvant decisions, unlike the NCCN chart.	**Adjuvant radiotherapy**recommended for patients with high-risk features such as positive margins, extracapsular extension, or multiple lymph node involvement. The goal is to reduce the risk of local recurrence.	**Surgical preference overemphasized**: Perplexity implies surgery is often recommended first, whereas NCCN promotes modality flexibility based on patient/tumor factors.
	≥2 positive lymph nodes without ENE; lymphovascular invasion or perineural invasion							
	**Indications for adjuvant chemoradiotherapy (CRT):** Extranodal extension (ENE); positive surgical margins.		**Neck management**:		**Adjuvant chemoradiation:** Following surgery, adjuvant chemoradiation is mandatory due to the high risk of recurrence.		**Adjuvant chemoradiotherapy:** For patients with high-risk features, adjuvant chemoradiotherapy (CTRT) is often advised.	**Adjuvant CRT indications overgeneralized**: Perplexity includes multiple positive nodes as CRT criteria - NCCN restricts CRT to ENE or positive margins only.
			**Definitive CRT approach**: Comprehensive RT fields including bilateral neck with boost to gross nodal disease					
			**Surgical approach**: MRND or comprehensive neck dissection (levels I–V). Bilateral neck dissection should be considered in: Midline primaries; primaries approaching midline; contralateral suspicious nodes; multiple					
			**Adjuvant CRT**: Standard of care for nearly all p16-negative patients with N2-N3 disease.					
p16-positive T0/2N0	**Initial treatment options**	**Clinical trials**: Omitted in ChatGPT - NCCN lists trials as a standard option for this group (category 2A).	**Initial treatment:**	**Clinical trials**: Not mentioned - NCCN lists them as valid options in this staging group.	**Initial Treatment:**	**Clinical trials**: Omitted — NCCN includes as valid treatment option in early p16+ cases.	**Initial treatment**	**Clinical trial omission**: Perplexity does not include clinical trials as a valid treatment option - NCCN does.
						**Neck management**: Omitted by Gemini - NCCN specifies selective neck dissection, including bilateral dissection for tongue base/midline tumors.		**CRT indications not clearly defined**: Perplexity refers vaguely to “multiple high-risk features,” which may imply CRT in broader contexts than NCCN allows (only ENE or margin+).
		**Re-resection as adjuvant option**: Not mentioned by ChatGPT - NCCN includes re-resection as an alternative to CRT when margins are positive.		**Adjuvant CRT**:		**Adjuvant CRT indication**: Gemini uses “multiple high-risk features”, which could be misinterpreted. NCCN restricts CRT to ENE or positive margins only.		**Re-resection** for positive margins omitted:
				- Omitted by Claude - NCCN explicitly recommends CRT for ENE or positive margin.				Perplexity doesn’t offer this alternative, which NCCN explicitly includes.
Question	**Transoral surgery (TORS or TLM) + observation or selective neck dissection.** In cN0 neck, elective selective neck dissection (levels II–IV) is often performed.	**Neck dissection detail**: ChatGPT doesn’t specify the need for bilateral neck dissection for tongue base tumors, which NCCN highlights.	**Primary tumor management**:	**Neck dissection**: Claude does not address bilateral dissection in tumors of the tongue base/midline, which is important per NCCN.	**Surgery (TORS or Open Surgery):**	**Re-resection**: Not offered by Gemini for positive margins - NCCN includes it as a category 2A option.	**Surgery:** Transoral surgery (e.g., transoral robotic surgery or laser microsurgery) is preferred when feasible. This approach is often combined with elective neck dissection to assess lymph node involvement.	**RT indications too narrow**: Perplexity restricts RT to cases with ENE or positive margins, ignoring intermediate-risk factors such as LVI, PNI, tumor size, and nodal characteristics that NCCN considers.
			Transoral surgery (TORS or TLM);		**Radiation Therapy (RT):** Definitive radiation therapy is a suitable alternative to surgery, especially in cases where surgery may result in significant functional impairment.			
			Definitive RT: Single-modality radiotherapy (60−66Gy)					
			**Neck Management**:		**Adjuvant Treatment:**		**Definitive RT**: For patients who are medically inoperable or prefer non-surgical treatment, definitive RT alone is sufficient for early-stage disease.	
			**With surgical approach**: Selective neck dissection (levels II-IV) recommended.		**Observation:** In patients with favorable pathologic features (e.g., negative margins, no extracapsular extension) following surgery, observation alone may be considered.			
			**With RT approach**: Elective nodal irradiation to ipsilateral neck (54Gy)					
What are the best options for initial and adjuvant treatment for the primary tumor and neck in patients with p16-positive oropharyngeal carcinoma T0-T2 N0?	**Radiotherapy alone**	**Risk stratification**: ChatGPT captures major features (ENE, margins, LVI/PNI) but misses others (e.g., nodal levels IV/V, tumor size > 3 cm, multiple nodes), important in NCCN decision-making.	**Adjuvant considerations**	**Adjuvant RT risk features**: Claude includes close margins and deep invasion but misses other features like LVI, nodal level IV/V, node > 3 cm.	**Adjuvant radiation therapy (RT):** Adjuvant RT is indicated for patients with adverse pathologic features, such as close or positive margins, or perineural invasion.	**Partial alignment on RT indications**: Gemini includes PNI and margin status but **misses other risk factors** listed by NCCN (e.g., LVI, nodal characteristics).	**Adjuvant RT**: Postoperative RT is recommended for patients with high-risk features, such as positive surgical margins or extracapsular extension.	**Bilateral neck dissection nuance missing**: Neck dissection is mentioned, but criteria for bilateral vs. ipsilateral approach are not specified, unlike NCCN.
	**Adjuvant therapy.** Often omitted in early-stage, p16-positive patients with favorable pathology (negative margins, no ENE, no LVI/PNI).		**Observation**: Appropriate for completely resected primary with negative margins and no adverse features.					
	If pathology shows intermediate risk features, adjuvant RT may be considered.		**Adjuvant RT**: Consider for close margins (< 3 mm), deep invasion, or presence of single positive node.		**Adjuvant chemoradiation (CRT):** Adjuvant CRT may be considered in cases with multiple high-risk pathologic features, particularly extracapsular extension.		**Adjuvant chemoradiotherapy (CRT)**: For patients with multiple high-risk features is recommended.	
	CRT is rarely needed in this group unless high-risk features are present – which is uncommon.							
p16-positive T0/2N1 (single node ≤3 cm)	**Initial Treatment**	**Missing treatment options**: ChatGPT does not include CRT (category 2B) or clinical trials, both recommended by NCCN.	**Primary tumor management**:	**Treatment options omitted**: Claude does not include CRT (category 2B) or clinical trials as NCCN does.	**Initial treatment:**	**Missing options**: No mention of clinical trials or concurrent CRT (category 2B) as valid first-line treatments — both are NCCN recommendations.	**Initial Treatment**	**Initial CRT promoted prematurely**: Perplexity lists concurrent CRT as default, while NCCN considers it a category 2B option, not standard for this early-stage setting.
				**Neck dissection detail**: Claude only mentions ipsilateral selective dissection, omitting bilateral dissection consideration based on tumor site.		**Neck dissection detail**: Gemini provides non-specific surgical detail, omitting criteria for ipsilateral vs. bilateral dissection.		**RT alone omitted**: Perplexity doesn’t mention RT as a standalone modality — NCCN lists it as a valid primary treatment.
		**Neck dissection details**: ChatGPT limits to ipsilateral dissection, but NCCN recommends bilateral dissection when anatomy indicates (e.g., midline tumors).	**Transoral surgery (TORS/TLM)**	**RT overuse in adjuvance**: Claude recommends RT for “most patients” post-surgery, while NCCN reserves adjuvant RT for defined intermediate-risk cases - some patients may avoid it.		**RT and CRT indications**: Partial match - Gemini includes some but not all risk features.		**Observation not included**: Perplexity skips observation, which NCCN allows in patients with clear margins and no adverse features.
						CRT is framed broadly (“multiple high-risk features”), while NCCN restricts it to ENE or margin+ only.		
Question	**Minimally invasive surgery (TORS or TLM) + Selective neck dissection:** Ideal when the tumor is accessible and resectable transorally.	**Re-resection option omitted**: Not offered in positive-margin scenarios - NCCN includes it alongside CRT.	**Definitive RT**: Single-modality RT (60−66Gy)	**CRT underemphasis**: Claude downplays standard CRT indications, implying that ENE or positive margins may not warrant CRT in p16+ cases — this diverges from NCCN, which maintains CRT as standard in these high-risk situations.	**Surgery (TORS or open surgery):** Careful surgical neck dissection	**Observation not included**: Observation omitted — NCCN allows no adjuvant therapy when margins are clear and no adverse features exist.	**Definitive radiotherapy (RT) with concurrent chemotherapy.**	**Adjuvant RT too restricted**: Perplexity only recommends RT for high-risk cases — misses intermediate-risk indications (e.g., close margins, LVI, multiple nodes).
	Elective neck dissection (levels II–IV) is performed ipsilaterally.							
	**Definitive radiotherapy (RT) alone.**		**Neck management (N1)**:		**Radiation therapy (RT):** Definitive radiation therapy is a viable alternative, especially if surgery is predicted to cause significant functional impairment.			
	**No adjuvant therapy if:** Negative margins							
	No ENE, LVI, or PNI							
	Single node ≤3 cm							
What are the best options for initial and adjuvant treatment for the primary tumor and neck in patients with p16-positive oropharyngeal carcinoma T0-T2, N1 (single node ≤3 cm)	**Adjuvant RT if:** Close margins or minor adverse features (LVI, PNI)	**Nodal-based risk features underexplored**: ChatGPT does not mention node > 3 cm, level IV/V, or multiple nodes, all considered in NCCN for adjuvant decision-making.	**With surgical approach**: Selective neck dissection (levels II–IV)	**Re-resection not mentioned**: NCCN offers it as an alternative in positive-margin cases - Claude does not.	**Adjuvant radiation therapy (RT):** Adjuvant RT is typically indicated if the surgical pathology reveals high-risk features, such as close or positive margins, or perineural invasion.	**Re-resection option not addressed**: Not offered, despite being listed by NCCN when margins are positive.	**Primary surgery**: Transoral resection of the primary tumor with ipsilateral or bilateral neck dissection	**Re-resection not discussed**: NCCN includes this as a management option for positive margins — Perplexity omits.
			**With RT approach**: Comprehensive ipsilateral coverage					
			**Adjuvant therapy. Observation**: May be appropriate for select cases with completely resected primary, single node without adverse features.					
	**Adjuvant CRT if:** Positive margins or ENE — rare in this scenario.		**Adjuvant RT alone**: Recommended for most patients (50−60Gy based on margin status).		**Adjuvant chemoradiation (CRT):** Adjuvant CRT might be considered if there are multiple high-risk pathologic features, especially extracapsular extension in the involved lymph node.		**Adjuvant radiotherapy (RT)**: Patients with high-risk features (e.g., positive margins, extracapsular extension) should receive adjuvant RT	
			**Adjuvant CRT**: Traditional indications (ENE, positive margins) may be less applicable for p16-positive disease.				**Adjuvant chemoradiotherapy (CRT)**: For patients with high-risk features, concurrent cisplatin-based CRT is recommended (EL I; Grade B).	
p16 (HPV)-positive T0/2-N1 (single node > 3 cm, or 2 or more ipsilateral nodes ≤6 cm) or T0/2N2 or T3N0/2	**Initial Treatment**	ChatGPT does not mention the option of induction chemotherapy followed by RT/CRT, which is classified as Category 3 in the NCCN guidelines.	**Primary Tumor Management**:	**A well-structured initial treatment plan:**	**Initial Treatment:**	**Initial treatment:**	**Initial Treatment**	**Partially aligned initial treatment:**
	**1. Surgery (TORS/TLM + Neck Dissection)**				**1. Definitive Chemoradiation (CRT)**		**1. Definitive Chemoradiation**	Induction followed by CRT is not mentioned.
	Bilateral or staged neck dissection may be indicated based on nodal burden and midline involvement.							Omitted clinical trials.
	**2. Definitive Radiotherapy (RT) ± Chemotherapy**				**2. Surgery**		**2. Surgery:** Transoral or open resection of the primary tumor with ipsilateral/bilateral neck dissection is an option for select cases, especially in early-stage disease.	Surgery is correctly included, but anatomical criteria for bilateral dissection are missing.
	**RT alone** may still be considered if total nodal burden is low and no adverse features are present.							
Question: What are the best options for initial and adjuvant treatment for the primary tumor and neck in patients with p16-positive oropharyngeal carcinoma T0-T2, N1 (single node > 3 cm or 2 or more ipsilateral nodes ≤6 cm) or T0- 2, N2 or T3, N0-2	**Concurrent CRT** is commonly used in patients with:	**Clinical trials** were not included as a treatment option.	**1. Definitive CRT**: Generally preferred for most patients in this category.	Indica corretamente CRT como abordagem padrão.	**Followed by Adjuvant Therapy:** The surgical treatment of the neck is very important for accurate staging, and treatment of the neck.	No clinical trials were mentioned.	**Adjuvant Treatment**	**Adjuvancy with slight excesses:**
	Bulky nodes		**2. Surgery for select cases**: Considered for resectable primaries where functional outcomes would be acceptable.	**There are no references to induction + CRT or clinical trials.**				
	T3 tumors		Consider bilateral neck treatment with:					
			Multiple nodal involvement					
	High concern for ENE (even radiologic suspicion)		Midline or approaching midline primaries					
			Large nodal disease (> 3 cm).					
	**Adjuvant Therapy After Surgery**		**Neck Management**:	**Well-founded cervical dissection:**	**3. Induction Chemotherapy:** Induction chemotherapy may be considered for bulky tumors to achieve downstaging before definitive local therapy (CRT or surgery).			CRT indicated for multiple lymph nodes, exceeding the NCCN guidelines.
	**RT alone if:**		**1. Definitive CRT approach**	Establishes clear criteria for a bilateral approach.				
	Negative margins		**Surgical approach**: 2. **Comprehensive neck dissection** (typically selective levels II-IV, with additional levels as indicated):	In line with the principles of the guideline.				
	No ENE							
	No high-risk features							
	Multiple nodes ≤6 cm without ENE							
	**CRT if:**		**Adjuvant Therapy**	**Adjuvant therapy with extended RT**: RT is recommended as "nearly universal," but the NCCN guidelines allow for observation of low-risk patients.	**Adjuvant Treatment:**	**Excessively aggressive adjuvancy:**	**1. Adjuvant Chemoradiation (CRT):** Recommended for high-risk features such as positive margins, extracapsular extension, or multiple positive lymph nodes	Observation does not appear as an option.
	ENE or positive margins		**1. Adjuvant RT**: Nearly universal in this patient group (50−60Gy)	**Properly indicated adjuvant CRT**: Administered only for ENE or a positive margin.	**1. Adjuvant Chemoradiation (CRT):** Following surgery, adjuvant CRT is strongly recommended.	CRT is recommended as the standard of care, even in cases without ENE or a positive margin—which goes beyond the NCCN recommendation.	**2. Adjuvant Radiotherapy (RT) Alone:** Used in cases with intermediate-risk features or when chemotherapy is contraindicated	Re-resection is not mentioned, although it is recommended by the NCCN in cases of positive margins.
	T3 primary with adverse pathology					Isolated RT is indicated only as an exception (QT intolerance), whereas the NCCN guidelines formally permit RT in intermediate-risk cases.		
	Radiologic ENE confirmed pathologically		**2. Adjuvant CRT**: Traditionally recommended for high-risk features (ENE, positive margins).	**Omission of re-resection**: Claude does not mention re-resection as an option for positive margins.	**Adjuvant Radiation Therapy (RT):** Adjuvant RT alone may be considered in patients who are unable to tolerate chemotherapy	Omission of the option for observation in low-risk cases.		
						Re-resection is not considered a viable option in cases of positive margins.		
p16-positive T0/3N3 or T4N0/3	**Initial treatment options**	**Initial treatment**: No clinical trials reported.	**Primary tumor management**:	**Partially aligned initial treatment:**	**Initial treatment:**	**Initial treatment**: No clinical trials reported.	**Initial Treatment**	**Initial treatment**
				Surgery is not mentioned, although it is particularly indicated for resectable T4 tumors.			**Definitive Chemoradiotherapy (CRT)**	Surgery is not mentioned, even though it is a valid option for resectable T4 tumors.
				Clinical trials also do not appear to be a viable option.			**Induction Chemotherapy**	Clinical trials were also omitted.
Question: What are the best options for initial and adjuvant treatment for the primary tumor and neck in patients with p16-positive oropharyngeal carcinoma T0/T3-N3 or T4-N0/N3	**Definitive chemoradiotherapy (CRT):** First-line in most cases	**Significant omissions**: Re-resection not mentioned.	**Definitive CRT**: Standard approach for most patients in this category.	**Complete lack of adjuvant therapy**: There is no discussion of adjuvant RT or CRT, nor of re-resection—this compromises the comprehensiveness of the management approach in surgical cases.	**Definitive chemoradiation (CRT):** Given the advanced nodal or T-stage, definitive CRT is often the cornerstone of treatment.	**Excessively aggressive adjuvant therapy**: Adjuvant CRT as a rule, regardless of pathological factors—this goes beyond NCCN guidelines, which restrict it to ENE or positive margins.	**Adjuvant Treatment**	**Adjuvância vaga e incompleta**: Faltam critérios de risco para definir RT ou CRT.
	**Surgery + adjuvant therapy (selected cases)**		**Induction chemotherapy (IC)** may be considered for bulky N3 disease		**Induction chemotherapy followed by definitive CRT:** Induction chemotherapy may be considered for very bulky tumors to achieve tumor downstaging before definitive CRT.	RT alone is only mentioned in cases where chemotherapy is contraindicated, not as a formal option for intermediate-risk cases.	**Radiation Therapy**	Re-resection and observation are not options.
	Reserved for resectable T4 tumors with acceptable functional outcomes (e.g., tongue base, lateral pharyngeal wall).							
	**Comprehensive neck dissection** required in N3 cases.		**Neck management**:		**Surgery followed by adjuvant therapy:** In select cases, surgery may be considered, but it often involves extensive resection and complex reconstruction.	It does not mention re-resection, a standard NCCN option for positive margins.		
	**Induction chemotherapy (not standard but investigated)** considered in very bulky disease.		**Definitive CRT approach**: Comprehensive bilateral RT fields covering primary and nodal disease.		**Adjuvant chemoradiation (CRT):** Following surgery, adjuvant CRT is mandatory due to the high risk of recurrence associated with advanced nodal or T-stage disease.	This observation does not appear, although it is rare in this group, but technically feasible.	**Chemoradiotherapy**	No mention of critical pathological factors underlying the decision in the NCCN guidelines.
	**Adjuvant therapy**		**Salvage surgery**: Reserved for: Residual disease after CRT; recurrent disease after primary non-surgical management.		Adjuvant Radiation Therapy (RT):			
	**1.** Adjuvant CRT is nearly always indicated in this group due to high-risk pathology.				**Adjuvant RT** alone may be considered in patients who are unable to tolerate chemotherapy			
	**2.** Adjuvant RT alone is rarely appropriate unless margins are negative, no ENE, and minimal nodal disease (unlikely in T4/N3).							
	High-risk pathology includes:							
	Extranodal extension (ENE)							
	Positive margins							
	Multiple nodes							
	T4 primary with adverse features.							

Analysis of the 28 generated responses revealed that 21 cases (75%) exhibited substantial congruence between LLM-generated recommendations and established guidelines, as confirmed through specialist consensus evaluation. Three instances showed significantly more comprehensive content within the guidelines compared to LLM outputs. Additionally, one response contained supplementary relevant information not included in the corresponding guidelines, whereas three responses presented direct contradictions to the guideline recommendations (Table 2). Table 3 delineates these findings stratified by individual LLM platforms. Chat GPT, Gemini and Perplexity LLMs presented one discordant answer each, in different questions. Platform Claude presented further information in one question, whereas the guideline presented more useful information in one question in comparison to Chat GPT, Claude and Perplexity.

**Table 2:** Direct contradictions to the guideline recommendations.

Question	Chat GPT	Claude	Gemini	Perplexity
p16-negative T1/2-N0/1	Discordant	Discordant	Discordant	Discordant
p16-negative T3/4a-N0/1	Guideline +	Guideline +	Guideline +	Guideline +
p16-negative T1/4a-N2/3	Guideline +	Guideline +	Discordant	Discordant
p16-positive T0/2-N0	Guideline +	Guideline +	Guideline +	Guideline +
p16-positive T0/2-N1	Guideline +	Guideline +	Guideline +	Guideline +
p16-positive T0/3N3 or T4N0/3	Guideline +	Guideline +	Guideline +	Guideline +

+, With relevant additional information.

**Table 3:** Findings stratified by individual LLM platforms.

	Chat GPT	Claude	Gemini	Perplexity
Similar content	5 (71.5%)	5 (71.5%)	6 (85.7%)	6 (85.7%)
Guideline with further content	1 (14.3%)	1 (14.3%)	0	0
Plataform with further content	1 (14.3%)	1 (14.3%)	1 (14.3%)	0
Discordant content	0	0	0	1 (14.3%)

## Discussion

This investigation examined the potential interface between AI systems and established medical guidelines. Medical guidelines function as a framework designed to preserve physician autonomy and clinical decision-making latitude, providing evidence-based recommendations applicable to the majority of patients rather than imposing mandatory protocols across all clinical scenarios.^10^ Over time, these guidelines have progressively been increasingly used as supporting evidence in legal proceedings. Table 1 presents the guideline recommendations, the corresponding formulated inquiries, the responses elicited from each LLM, and the substantive deviations from the original recommendations.

The expanding role of LLM within clinical medicine has elicited significant interest among researchers, necessitating comprehensive exploration of their potential applications and inherent limitations. Despite the existing body of literature examining AI applications in Otolaryngology, only recently have comparative analyses emerged evaluating the relative capabilities of various LLMs within this specialized medical domain.^11^

This investigation is distinguished by its comparative analysis of LLM and represents one of the initial efforts to employ externally validated assessment instruments specifically designed for otolaryngology applications. The methodological decision to utilize two distinct quality assessment tools for evaluating LLM performance was predicated on the nuanced differences between the specific domains these instruments examine.^12^

When analyzing the cases in which AI-generated recommendations diverged from the NCCN guidelines, we observed that these discrepancies may have relevant clinical implications. In scenarios of locally advanced disease, for example, some platforms suggested management strategies that could lead to overtreatment, such as prematurely recommending more aggressive surgical approaches, or to undertreatment, by minimizing the need for combined therapeutic modalities. These divergences reinforce that the use of AI in clinical decision support still requires caution, particularly in complex situations where small variations in treatment strategy may directly impact oncologic control, functional preservation, and patient quality of life. Therefore, we emphasize that AI should be interpreted as a complementary tool to specialized clinical judgment, and not as a substitute for the expertise of the treating specialist.

The management of patients with Oropharyngeal Squamous Cell Carcinoma (OPSCC) remains contentious, as no definitive randomized trials have adequately addressed the comparative efficacy of surgical intervention, Radiation Therapy (RT), or multimodal treatment approaches. Contemporary understanding recognizes HPV-associated OPSCC as a distinct clinicopathological entity from HPV-negative disease, characterized by differential epidemiological distribution patterns and prognostic outcomes. Given the superior clinical outcomes observed in HPV-positive patients compared to their HPV-negative counterparts, HPV-positive OPSCC may warrant more individualized therapeutic strategies designed to mitigate late sequelae induced by surgical intervention, radiotherapy, and systemic therapies. Various de-intensification approaches have been investigated with the objective of delivering less toxic treatment regimens while maintaining comparable disease control and survival metrics.^13^

It is imperative to emphasize that the specificity of responses generated by LLM is fundamentally contingent upon the precise construction and formulation of inquiries. Accordingly, the methodological section provides detailed documentation of the specific prompts employed in this investigation, ensuring both procedural transparency and experimental reproducibility.

A comparative examination of guideline development methodologies versus AI implementation for clinical recommendation generation reveals significant temporal and procedural distinctions. Guideline development necessitates considerable temporal investment and requires collaborative participation from multiple domain specialists who conduct systematic reviews of contemporary literature on specific clinical domains. Following publication, guidelines face progressive obsolescence as emerging evidence accumulates, remaining outdated until subsequent revision cycles occur. Conversely, AI systems deliver real-time responses with the potential to maintain currency with evolving evidence, contingent upon the specific platform’s knowledge base update frequency and implementation parameters.

AI demonstrates significant potential to augment numerous aspects of oral cancer management through its progressive integration into clinical practice. The development of clinical guidelines constitutes a resource-intensive and financially substantial process, with revision cycles typically occurring at multi-year intervals. During these extended periods, considerable scientific advancements and knowledge evolution may transpire. LLM presents the distinct advantage of enabling immediate consultation capabilities, thereby facilitating access to contemporaneous evidence-based information.

The potential role of LLMs in clinical practice presents an interesting dichotomy ‒ they may serve either as adjunctive resources or as alternatives to traditional clinical guidelines. Healthcare practitioners could employ these technologies at the point of care for expedient information retrieval on guideline-covered topics. Effective utilization would necessitate the development of tailored prompts with sufficient contextual information and precise inquiries, followed by critical evaluation to remove unnecessary elements from the generated responses. Despite these requirements, the clinical value of such technology-assisted information remains significant. These systems provide recommendations that reflect contemporary understanding at the time of inquiry. In contrast, formal clinical guidelines typically undergo periodic updates at extended intervals, while medical knowledge exists in a state of continuous flux. This scientific evolution is accelerated in contemporary healthcare, where new evidence emerges with remarkable frequency. This temporal disparity may result in guidelines that do not incorporate the most recent scientific developments. Although the concordance rate observed is encouraging, it is essential to consider that the remaining 25% represents a significant potential risk if AI recommendations are used autonomously. Thus, we reinforce that AI should function as a complementary support tool in the decision-making process, and not as a substitute for medical expertise.

A limitation of this study is the inclusion of only four commercially available LLMs. Although these platforms were selected because, at the time of data collection, they presented broader and more accessible medical knowledge bases, this choice may restrict the generalizability of our findings to other AI systems or future versions of the same models. In addition, these tools were not originally developed as dedicated clinical decision-support systems, which may have contributed to some of the discrepancies observed in relation to established guidelines. A significant methodological limitation of this investigation is that the comparative assessment between guideline content and LLM-generated responses potentially introduced evaluative bias attributable to subjective interpretative frameworks employed by experts when determining the clinical significance of discrepancies between these information sources. The absence of validated evaluation methodologies suggests that results could vary considerably with different assessment instruments, potentially leading to inconsistencies in artificial intelligence technology evaluation. This methodological variability could substantially influence the adoption of and trust in AI systems within the medical community. Consequently, additional research is warranted to establish standardized, reproducible methodologies for AI evaluation, ensuring that these technologies can be reliably integrated into clinical practice to enhance patient care outcomes. The incorporation of such methodologies into subsequent investigations could significantly advance our understanding of AI performance in clinical contexts, thereby facilitating more robust integration of AI tools in medical practice. As a natural continuation of this research, we intend to expand the scope of future investigations by incorporating additional platforms, including academic LLMs and models specifically designed for clinical decision support.

## Conclusion

The findings of this study indicate that, with optimized query formulation, LLMs achieved a remarkable 75% concordance with established guideline recommendations. This degree of concordance suggests promising potential for LLM to augment clinical decision-making processes in practical settings. However, comprehensive validation studies remain essential to rigorously authenticate the accuracy and reliability of LLM-generated responses prior to their full integration into clinical applications. Consequently, at this developmental stage, it is prudent to conceptualize these AI systems as valuable complementary instruments rather than replacements for established clinical guidelines. Their functional role should be to enhance, rather than supplant, clinician judgment, thereby ensuring that patient care remains fundamentally grounded in reliable, evidence-based practices.

## ORCID IDs

Mario Augusto Ferrari de Castro: 0000-0003-0234-9807

Leandro Luongo de Matos: 0000-0002-5068-8208

Caio Paschoalin Trindade: 0009-0003-8408-3180

Bruno Pelinson Fogaça Duarte: 0000-0001-6667-1526

Luiz Paulo Kowalski: 0000-0002-0481-156X

## Funding

None.

## Data availability statement

The authors declare that all data are available in repository.

## Declaration of competing interest

The authors declare no conflicts of interest.
